# Dexmedetomidine Versus Midazolam for End-of-Life Sedation and Agitation: Protocol for a Randomized Controlled Trial (The DREAMS Trial)

**DOI:** 10.2196/55129

**Published:** 2024-09-04

**Authors:** Benjamin Thomas, Greg Barclay, Wing-Shan Angela Lo, Judy Mullan, Kylie Mansfield

**Affiliations:** 1 Graduate School of Medicine University of Wollongong Wollongong Australia; 2 Palliative Care Service Illawarra Shoalhaven Local Health District Wollongong Australia

**Keywords:** palliative, sedation, delirium, dexmedetomidine, midazolam, antipsychotics, deep sedation, palliative care, sedative, adult, inpatient, Australia, quality of life, end of life, protocol, dexmedetomidine for the reduction of end-of-life agitation and for optiMised sedation, DREAMS trial, DREAMS, deep sedation

## Abstract

**Background:**

Sedation at the end of life is used to relieve distressing symptoms including agitation and delirium. Standard care may include infused benzodiazepines or antipsychotics. These agents often result in deep sedation with loss of interaction with loved ones, which may be distressing.

**Objective:**

The DREAMS (Dexmedetomidine for the Reduction of End-of-life Agitation and for optiMised Sedation) trial aimed to compare the sedative and antidelirium effects of the alpha-2 agonist dexmedetomidine, a novel palliative care sedative, compared with midazolam, a benzodiazepine when administered by subcutaneous infusion at the end of life, with doses of both agents targeting lighter, or potentially interactive sedation.

**Methods:**

Participants were recruited from adult inpatients admitted for end-of-life care under a palliative care team in regional New South Wales, Australia. Inclusion criteria included patients older than 18 years, with a preference for lighter sedation at the end of life. Exclusion criteria included severe cardiac dysfunction (contraindication to dexmedetomidine). Participants consented and were placed on a treatment-pending list. Upon experiencing terminal deterioration, patients were randomized to either arm 1 (dexmedetomidine) or arm 2 (midazolam) as their treatment arm. These treatments were administered by continuous subcutaneous infusion. The level of consciousness and agitation of the patients were measured by the Richmond Agitation-Sedation Scale–Palliative version and the Memorial Delirium Assessment Score. Richmond Agitation-Sedation Scale–Palliative version assessments were performed by both nursing and medical staff, while Memorial Delirium Assessment Score assessments were carried out by medical staff only. Families and patients were asked to complete, as able, a patient comfort assessment form, to gauge perceptions of distress. Data were collected and matched with the breakthrough medication doses administered, along with qualitative comments in the medical record. In addition, the study tracked symptoms and patient functional status that were recorded as part of the Palliative Care Outcomes Collaborative, a national tracking project for monitoring symptom outcomes in palliative care.

**Results:**

The DREAMS trial was funded in May 2020, approved by the ethics committee in November 2020, and started recruiting participants in May 2021. Data collection commenced in May 2021 and is anticipated to continue until December 2024. Publication of results is anticipated from 2024 to 2026.

**Conclusions:**

The evidence base for sedative dosing in palliative care for distress and agitation is not robust, with standard care based primarily on clinical experience and not robust scientific evidence. This study is important because it will compare a standard and a novel sedative used in end-of-life treatment. By assessing the potential efficacy and benefits of both, it seeks to optimize the quality of dying by providing targeted sedation that can improve the communication between dying patients and their loved ones.

**Trial Registration:**

Australia New Zealand Clinical Trials Register ACTRN12621000052831; https://uat.anzctr.org.au/Trial/Registration/TrialReview.aspx?id=380889

**International Registered Report Identifier (IRRID):**

DERR1-10.2196/55129

## Introduction

### Background

Palliative care includes the holistic, person-, and family-centered care provided for patients with a life-limiting diagnosis where the primary goal is to optimize quality of life. At the end of life, patients may experience hardship from an array of distressing symptoms including pain, nausea, agitation, and distress, with up to 88% of patients admitted to a palliative care unit enduring terminal delirium [1]. When targeted symptom management is unsuccessful and patients are distressed, the most appropriate therapy offered may be sedation to relieve these symptoms [2-4] The current standard of care for distress at the end of life includes infused benzodiazepines and antipsychotics, or infused barbiturates in refractory cases [5,6]. In Australia, the injectable benzodiazepine, midazolam, is the typical first-line sedative used, which is in line with the position statement of the Australian and New Zealand Society of Palliative Medicine (ANZSPM) [5] and is consistent with recommendations from the European Association for Palliative Care (EAPC) framework for sedation at the end of life [6].

What constitutes comfort at the end of life is variable, but freedom from pain and severe symptoms along with clear communication is valued by patients and families [7,8]. Patients requiring end-of-life sedation to control their symptoms may appear externally comfortable to their treating clinicians. However, the loss of their biographical life and interactivity is often difficult and distressing to their families and loved ones, as well as to the patients themselves [9]. The use of alternative sedatives to relieve end-of-life distress or delirium that may allow meaningful interaction may be considered desirable if symptoms remain controlled.

There is currently poor-quality evidence for the use of midazolam at the end of life. Doses are based on the outcomes from a total of 319 patients, reported across a series of small case series [10-14], 1 multicenter study [15], and 1 prospective study [16]. There is no evidence base presented for the doses chosen, with usage predominantly predicated on professional consensus and guidelines [5,6]. Despite this, the use of midazolam is recommended as the first-line therapy by palliative care specialist society consensus [5].

Dexmedetomidine is an imidazole alpha-2 receptor agonist commonly used in intensive care and anesthetic settings [17,18]. It exhibits activity in the central nervous system, which includes decreasing sympathetic activation at the locus coeruleus [19]. Patients sedated with dexmedetomidine may achieve a state resembling non–rapid eye movement sleep, may be easily woken, and may interact with often minimal stimulation, as well as a experience a decrease in delirium [18]. Dexmedetomidine was investigated in a pilot clinical trial at the Port Kembla palliative care unit (PKPCU) for agitated delirium at the end of life, with results showing a trend toward interactive sedation and decreased delirium [20].

Dexmedetomidine, outside of the recent pilot trial, has been used in the palliative care setting for intractable pain [21,22], refractory nausea and vomiting [23], and sedation [24,25]. In addition, there is an ongoing clinical trial in palliative medicine investigating dexmedetomidine for delirium (ClinicalTrials.gov NCT04824144). It is currently approved in Australia and the United States for administration at doses of up to 0.7 µg/kg/h by infusion [26]. In intensive care unit settings, its usage has often exceeded this dosage level without significant side effects [16,17]. Traditionally given as an infusion, dexmedetomidine has also been shown to be safely tolerated as a rapid intravenous bolus without significant hemodynamic compromise [27,28].

Dexmedetomidine is also well-tolerated subcutaneously with attenuated side effects [29] and a very low incidence of injection-site reactions [20]

Comparative literature on dexmedetomidine and midazolam is limited, with only a single entry in clinical trial registries (ClinicalTrials.gov NCT01687751). The trial, registered in 2012, focused on using rapidly titrating continuous subcutaneous infusion (CSCI) and was withdrawn before recruitment due to feasibility issues.

Overall, there is limited robust evidence to guide the use of dexmedetomidine or midazolam as end-of-life sedatives in the palliative care population.

### Objectives

Given the knowledge gap, the investigators proposed a randomized controlled trial, “Dexmedetomidine for the Reduction of End-of-life Agitation and for optiMised Sedation” (DREAMS). This trial aimed to investigate the use of dexmedetomidine versus midazolam by CSCI in patients for sedation at the end of life. The subcutaneous route was chosen for the DREAMS trial as the preferred route of delivery as this conforms to the current standard of care for medication infusions given to patients under palliative care in Australia [4].

### Hypothesis

Dexmedetomidine provides more interactive sedation at the end of life while maintaining comfort when compared with midazolam.

### Primary Objective

The primary objective of this study was to determine if dexmedetomidine is superior to midazolam arousability without agitation as measured by the Richmond Agitation-Sedation Scale–Palliative version (RASS-PAL) [30] in the terminal phase of life.

### Secondary Objectives

The key secondary objectives are to determine if dexmedetomidine, for terminal patients, is superior compared with midazolam for (1) reduction in delirium as measured by the Memorial Delirium Assessment Scale (MDAS) [31], (2) reduction in the use of breakthrough medications for symptom burden, and (3) improving perceptions of comfort by families and loved ones as measured by patient comfort assessment (PCA) form [32].

The DREAMS trial is designed as a randomized, controlled, nonblinded multicenter superiority trial with 2 parallel groups and a primary end point of average daily RASS-PAL score during the terminal phase. Randomization will be performed as block randomization, with a 1:1 allocation.

## Methods

### Trial Setting

The trial was conducted with patients under the care of the palliative care service in the Illawarra Shoalhaven Local Health District in New South Wales, Australia. It was conducted within the palliative care units located across multiple sites, including Port Kembla Hospital, The Wollongong Hospital, and Shoalhaven Memorial District Hospital. Each of the 3 sites was chosen pragmatically with the allocation made of numbers based on their historical referral base. The trial was sponsored by the Illawarra Shoalhaven Local Health District Research Office.

### Ethical Considerations

#### Human Participant Ethics Review Approvals

The DREAMS trial received ethical approval from the joint University of Wollongong (UOW) and Illawarra Shoalhaven Local Health District Human Research Ethics Committee in November 2020 (approval 2020/ETH01943). Research was conducted in line with the National Statement on Ethical Conduct in Human Research, Australia.

#### Informed Consent

All participants approached in the DREAMS trial provided informed consent. This process included the provision of the participant information sheet, opportunities for discussion with investigators and families, and the assurance that participants could withdraw at any point from the trial. Consent directly from the participant was preferred. However, consent provided by a responsible person (proxy), as permitted under NSW Law [33], was deemed acceptable but not preferred.

#### Privacy and Confidentiality

All data collected were deidentified before analysis. Preanalysis data are stored in encrypted cloud storage within the NSW Health and UOW networks, with hard copies of data retained within secure health storage.

#### Compensation

No compensation was provided for participation in the DREAMS trial.

### Eligibility Criteria

Participants were considered for recruitment if they met the inclusion and exclusion criteria (Textbox 1).

**Inclusion and exclusion criteria.**

**Inclusion criteria**

- Admitted to hospital under a palliative care physician, for end-of-life care.
- English speaking or able to converse through a medical interpreter.
- Adults aged 18 years or older.
- Preference or acceptance of lighter sedation at the end of life expressed by the participant during consent discussions and questions around the trial.

**Exclusion criteria**

- Known severe left ventricular dysfunction (defined as an ejection fraction of <20% on known echocardiography), in patients with a previous history of heart failure, as available within the past 12 months either by inpatient or outpatient scans.

Participants who consented to take part in the DREAMS trial remained enrolled until death or until choosing to withdraw from participation. If participants did not meet the initiation criteria and therefore could not be randomized, they were automatically withdrawn from the study.

### Randomization and Initiation

Participants were randomly assigned to either dexmedetomidine or midazolam through blocks of 6 in a 1:1 allocation as per a computer-generated randomization schedule. Randomization was based on total patient recruitment and was not site-specific. Assignment occurred when participants were determined to be appropriate for treatment by a senior clinician. Appropriateness was determined on 2 criteria—a clinician’s prediction of survival of 7 days or less and the presence of symptoms of distress requiring treatment with sedative medications at the end of life.

### Interventions

Eligible patients were randomized in equal proportions to receive either dexmedetomidine or midazolam. Both drugs, in generic formulations, were purchased through the contract supplier for NSW Health. The doses were targeted based on weight and administered by CSCI using a NIKI T34 syringe pump (CME Biomedical Services) [34]. *Pro re nata* (PRN) subcutaneous bolus injection doses were also available and calibrated by weight with reference to literature as well as previous practice [26,35-37] and the previous trial at the PKPCU [20]. Doses were calculated as per Table 1.

**Table 1:** Doses of dexmedetomidine and midazolam given per trial. All dexmedetomidine doses were rounded up to the nearest 10 µg, and midazolam infusion doses were rounded up to the nearest 1 mg.

Administration	Dexmedetomidine	Midazolam
Infusion	0.5 µg/kg/h	0.25 mg/kg/d
Breakthrough	0.5 µg/kg every 2 hours	2.5-5 mg every 2 hours

Infusion doses were rounded to the nearest 10 µg (dexmedetomidine) or 1 mg (midazolam) due to the practicalities of ward-based administration, as were dexmedetomidine breakthrough doses. Midazolam breakthrough doses were not rounded up or down. Requirements for breakthrough medications were clinically determined by staff members caring for individual patients in response to symptoms of agitation and distress that did not spontaneously resolve and were distressing to patients or family members. Breakthrough doses were limited to a maximum of 5 doses daily to ensure expedited clinical review if needs exceeded availability. For patients who were not benzodiazepine-naïve, an equivalent use of their previous benzodiazepine [37], converted to midazolam, was added to their calculated dose.

**Figure 1:**
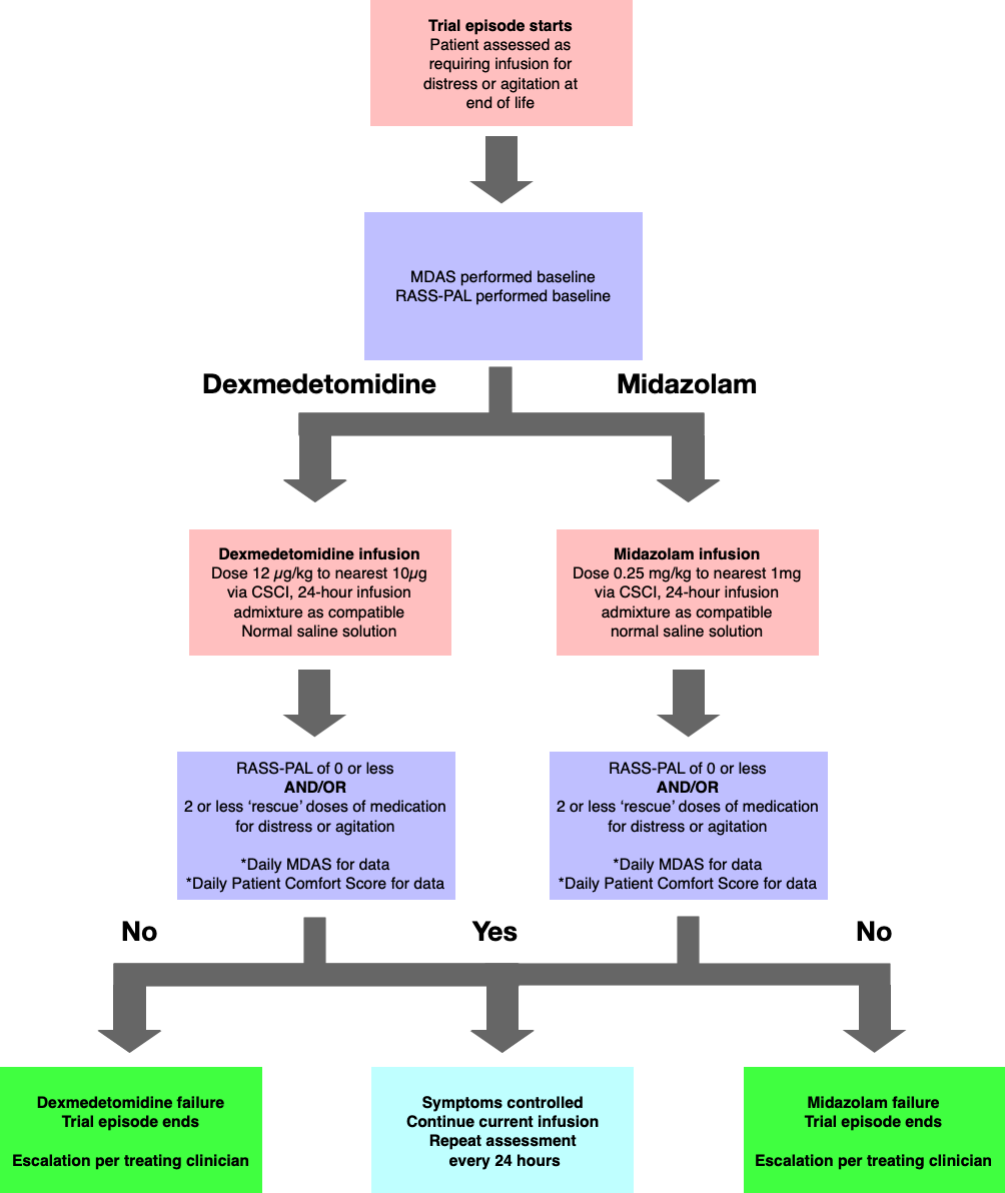
Trial daily run. MDAS: Memorial Delirium Assessment Scale; RASS-PAL: Richmond Agitation-Sedation Scale–Palliative version; CSCI: continuous subcutaneous infusion.

Both midazolam and dexmedetomidine were administered using separate infusion devices to other medications (although in practice both are compatible in admixture) to eliminate potential reactions. Daily intervention delivery and assessments were performed as per Figure 1.

Patients were administered a RASS-PAL and an MDAS on initiation by the treating medical staff, and then a RASS-PAL once per shift by nursing staff. The daily average RASS-PAL score was collated, with a target of 0 or below. If patients remained comfortable and the RASS-PAL score was in the appropriate range, or there had not been a high use of rescue PRN medication for distress (defined as 3+ doses of either dexmedetomidine or midazolam, without other rationale), infusions continued with daily MDAS and regular RASS-PAL assessments. For patients who were uncomfortable, or who had an inappropriate range of RASS-PAL scores or PRN use, trial episodes were ceased, and treatment was continued as per the admitting specialist’s clinical discretion, in line with the ANZSPM and EAPC frameworks [5,6].

Regular training sessions were held with staff members involved in the trial. The chief investigator provided group and one-to-one education with nursing and medical staff, which were repeated during the trial run at all involved sites. Regular education was scheduled for the primary site at Port Kembla Hospital, with opportunistic education around recruitment performed for the smaller sites. Training included discussion of tools, medications, dosing, side effects, and adverse reaction reporting.

### Sample Size

The sample size was calculated based on the primary outcome. Based on an expected between-group difference of 1 unit on the RASS-PAL (10% difference) with an SD of 1.26, it was estimated that each study arm would require 26 patients (52 in total) to achieve statistical significance. This calculation, targeting an α of .05 and 80% power, was performed by the Statistical Consulting Centre, UOW based on an independent sample 2-tailed *t* test for between-group differences.

### Recruitment

Each site screened participants according to the inclusion and exclusion criteria (Textbox 1), with varying recruitment targets based on referral rates (40 patients in Port Kembla Hospital, 8 patients in Wollongong Hospital, and 4 patients in Shoalhaven Hospital). The enrollment period initially planned to take place over 18 months, was impacted significantly by the COVID-19 pandemic, leading to an extension of 32-month duration as well as a heavier focus on recruitment at Port Kembla Hospital due to recruitment difficulties at the other sites.

Baseline characteristics of consented patients were collected, including sex, primary diagnosis, background diagnoses, weight, and liver and kidney function. Liver function was assessed by the model of end-stage liver disease-sodium (MELD-Na). Kidney function was assessed by estimated glomerular filtration rate (eGFR). MELD-Na and eGFR were calculated based on the most recent blood tests taken within the past 3 months upon entry into the trial. If no recent blood test was performed, a biochemical profile including serum sodium, bilirubin, creatinine, and international normalized ratio was obtained to perform dose calculations of MELD-Na and eGFR. Weight was used to calculate doses of both dexmedetomidine and midazolam in the event of randomization.

### Data Collection and Management

#### Primary Outcome

Agitation and sedation were measured using the RASS-PAL on all patients on initiation of trial phase, and regularly by nursing and medical staff during their treatment episodes. The RASS-PAL is a 10-point scale, ranging from –5 (completely sedated) to +4 (significant agitation), with a score of 0 indicating alert and calm. The Richmond Agitation-Sedation Scale (RASS) has excellent interrater reliability and has been used in previous studies in palliative care and consciousness [38]. The palliative adapted version of the Richmond Agitation-Sedation Scale (ie, RASS-PAL) was used due to its appropriateness in the palliative care setting, and previous use in palliative care [20,30]. The RASS-PAL was performed every shift by nursing staff, and daily by medical staff.

#### Secondary Outcomes

Delirium was monitored using the MDAS, which has been validated in cancer delirium and the palliative care population, as well as delirium treated with dexmedetomidine [1,20,31,39]. The MDAS is a 10-item, 4-point clinician-rated scale that is designed to quantify the severity of delirium in medically ill patients [34]. Some patients required pro-rata scoring of the MDAS due to the inability to complete the full item list; this scoring was done in line with the instruction per instrument [31]. Comfort was assessed by patients and carers using the validated PCA [32], which assesses comfort on a 1-10 scale with family comments, as well as recording ratings for specific symptoms including grimacing, groaning, shouting, movements, and labored breathing [32]. Data regarding symptom severity, associated distress, and breakthrough medications administered as well as potential effectiveness are routinely collected and documented in the patient’s electronic health record (EHR). These data are also used for reporting and analysis by the Palliative Care Outcomes Collaboration (PCOC), an Australian partnership providing longitudinal data on palliative care outcomes across various sites and services [40]. Palliative Care Outcomes Collaborative data will be matched with the PCA tools collected, facilitating a comparison of family, patient, and staff perceptions of comfort. These will also be correlated to breakthrough and background medication doses as documented in the EHR to assess potential effects.

#### Data Management

The RASS-PAL, MDAS, and PCA were collected as hardcopy printouts and scanned and stored in secure cloud storage in the NSW Health and UOW networks. EHR data will be extracted and stored in encrypted spreadsheets and within secure cloud storage. Data will be retained for a minimum of 15 years after publication of study results, as per the NSW State Archives and Records Guidance GDA17.

### Statistical Methods

The dexmedetomidine arm and the midazolam arm will be compared against one another for all primary analyses. The authors will also perform intra-arm analysis for day-to-day differences in each treatment arm for the MDAS analysis and the RASS-PAL analysis. Binary outcomes will be assessed by chi-square and continuous outcomes by the 2-tailed *t* test. *P* values will be reported to four decimal places, with values less than .001 reported as <.001. We will use 2-tailed *P* values with an α<.05 level of significance.

Descriptive statistics will be used where data are not amenable to the above analysis.

### Monitoring

A formal data monitoring committee was not established due to the relatively low-risk nature of the study and population. Oversight was provided by 2 senior clinicians associated with the PKPCU, who reviewed interim analysis of the data after the first 10 patients were recruited and were appraised of adverse effects as the trial progressed.

The DREAMS trial used reporting as per Common Terminology Criteria for Adverse Events for adverse event reporting [41]. Minor adverse events deemed secondary to either study drug were recorded by medical staff and will be collated for the therapeutic regulator. Major adverse events requiring immediate notification were planned to result in immediate trial episode infusion for affected patients, reversal of adverse effects if clinically appropriate, and reporting to the oversight team and the regulator.

During the clinical phase of the trial, there was only 1 adverse outcome reportable to the oversight team, consisting of an accidental excess breakthrough dose administration of 1 of the trial medications, which resulted in no harm and no adverse outcome to the participant in question.

## Results

The study was funded by an Early Career Research Grant from the Illawarra Shoalhaven Local Health District Research Office in May 2020, with ethics approval granted in November 2020. Participant enrollment began in May 2021 and completed in November of 2023. Consent was obtained from 58 patients in total, with only 1 patient declining consent when the trial was discussed. Of the 58 consented patients, 52 in total were treated, 26 per arm, with 6 patients not requiring treatment with a trial medication due to not developing symptoms of agitation or distress in their dying phase.

Data collection is expected to continue until December of 2024. Data analysis is anticipated to begin in mid-2024, with initial results anticipated to be reported on and published after the completion of data collection. Ongoing publications based on ongoing analysis and collection are anticipated to continue in 2025 and 2026.

## Discussion

### Principal Findings

The DREAMS trial investigators aim to test the efficacy of a novel sedative versus a standard care sedative at the end of life with targeted dose protocols, with the goals of maintaining comfort while improving interactivity and ability for dying patients to interact with their loved ones and thus maximize the quality of dying. It is hypothesized that there will be an observed difference in both sedation and delirium. The protocol for the DREAMS trial has been designed to balance the need for rigorous scientific investigation with the flexibility and pragmatism involved in the provision of end-of-life care to a vulnerable population [42,43]. The trial aims to compare the effects of dexmedetomidine against midazolam through subcutaneous infusion with doses targeting lighter sedation to assess the acceptability and efficacy of both, with a hypothesis to the superiority of dexmedetomidine in maintaining arousability as measured by RASS-PAL. The alleviation of symptoms of pain, agitation, and distress at the end of life will remain paramount, with pragmatic titration of other medications for symptoms provided as per best practice [37,44], to ensure that individual patient needs remain fulfilled.

### Strengths and Limitations

This study is notable in being the first randomized controlled trial of midazolam in the palliative care setting, as well as the first to compare novel and traditional sedatives. Significant potential exists to impact the standard of care based on this study’s findings, given the lack of robust previous data. The study’s methods are limited by a lack of blinding, which was due to a lack of funding for research staff as well as unpredictable time frames and admission numbers that were exacerbated by the COVID-19 pandemic. Randomization by computer-generated allocation was intended to help alleviate bias in treatment provision. Logistical barriers to ongoing education for nursing and medical staff completing trial assessments were identified, with staggered shifts necessitating recurring education to capture the whole cohort of assessors, as well as rotational and casual staff members requiring frequent refresher training. The lack of validated midazolam dosing in palliative care resulted in extrapolation and calculation from the anesthetic literature as well as standard practice [14,36,37].

If the dexmedetomidine intervention proves superior to midazolam, it may result in an increase in the provision of and use of that agent at end-of-life care. Conversely, if midazolam proves noninferior, assuming both agents provide comfort and symptom relief, this could validate both the standard care arm and the novel arm as potentially useful tools for the treatment of symptoms. Analysis of the patient-centered data including the PCA forms and qualitative comments would be of paramount importance then to help discriminate between options, and to aid in improving and optimizing the quality of dying for patients at the end of life.

## Appendix

Multimedia Appendix 1 CONSORT Checklist.

## Abbreviations

ANZSPM: Australian and New Zealand Society of Palliative Medicine
CSCI: continuous subcutaneous infusion
DREAMS: dexmedetomidine for the reduction of end-of-life agitation and for optiMised sedation
EAPC: European Association for Palliative Care
eGFR: estimated glomerular filtration rate
EHR: electronic health record
MDAS: Memorial Delirium Assessment Scale
MELD-Na: model of end-stage liver disease-sodium
RASS: Richmond Agitation-Sedation Scale
PCA: patient comfort assessment
PCOC: Palliative Care Outcomes Collaboration
PKPCU: Port Kembla palliative care unit
PRN: pro re nata
RASS-PAL: Richmond Agitation-Sedation Scale–Palliative version
UOW: University of Wollongong

## Data Availability

The data sets generated during and/or analyzed during this study will be available from the corresponding author on reasonable request, with appropriate consent from the ethics committee for potential sensitive data release if required.
